# Perceived Stress at Work Is Associated with Lower Levels of DHEA-S

**DOI:** 10.1371/journal.pone.0072460

**Published:** 2013-08-28

**Authors:** Anna-Karin Lennartsson, Töres Theorell, Alan L. Rockwood, Mark M. Kushnir, Ingibjörg H. Jonsdottir

**Affiliations:** 1 The Institute of Stress Medicine, Gothenburg, Sweden; 2 The Department of Neuroscience and Physiology, University of Gothenburg, Gothenburg, Sweden; 3 Stress Research Institute, Stockholm University, Stockholm, Sweden; 4 ARUP Institute for Clinical and Experimental Pathology, Salt Lake City, Utah, United States of America; 5 Department of Pathology, University of Utah School of Medicine, Salt Lake City, Utah, United States of America; University of Jaén, Spain

## Abstract

**Background:**

It is known that long-term psychosocial stress may cause or contribute to different diseases and symptoms and accelerate aging. One of the consequences of prolonged psychosocial stress may be a negative effect on the levels of dehydroepiandrosterone (DHEA) and its sulphated metabolite dehydroepiandrosterone sulphate (DHEA-S). The aim of this study is to investigate whether levels of DHEA and DHEA-S differ in individuals who report perceived stress at work compared to individuals who report no perceived stress at work.

**Methods:**

Morning fasting DHEA-S and DHEA levels were measured in serum in a non-stressed group (n = 40) and a stressed group (n = 41). DHEA and DHEA-S levels were compared between the groups using ANCOVA, controlling for age.

**Results:**

The mean DHEA-S levels were 23% lower in the subjects who reported stress at work compared to the non-stressed group. Statistical analysis (ANCOVA) showed a significant difference in DHEA-S levels between the groups (p = 0.010). There was no difference in DHEA level between the groups.

**Conclusions:**

This study indicates that stressed individual have markedly lower levels of DHEA-S. Given the important and beneficial functions of DHEA and DHEA-S, lower levels of DHEA-S may constitute one link between psychosocial stress, ill health and accelerated ageing.

## Introduction

It is known that long-term psychosocial stress may cause or contribute to different diseases and symptoms [1]–[3] and accelerate aging [4], [5]. One of the consequences of prolonged psychosocial stress may be a reduction of the levels of dehydroepiandrosterone (DHEA) and its sulphated metabolite dehydroepiandrosterone sulphate (DHEA-S). DHEA and DHEA-S are, a long with cortisol, produced in the adrenal cortex in response to adrenocorticotropic hormone (ACTH). Cortisol and DHEA are produced in different sections of the adrenal cortex; the zona fasciculata area secretes cortisol while the zona reticularis area secretes DHEA and DHEA-S [6]. In addition to their role as sex steroid precursors, DHEA and DHEA-S are anabolic androgens and thus they have a protective and regenerative role [7], [8]. Levels of DHEA and DHEA-S are age dependent; peak levels are reached in early adulthood and decline thereafter [9]. DHEA and DHEA-S have been shown to be associated with a wide range of health outcomes. Low levels of DHEA and DHEA-S have been associated with different disease states, such as for example depression [10], low-back pain and slow rehabilitation of low-back pain in women [11]–[13] and mortality of cardiovascular disease in elderly men [14] while high DHEA-S levels have been associated with good health and well-being [11]. DHEA and DHEA-S levels vary also in healthy individuals of same age, depending on genetics [15] and likely environmental and life style factors. Long-term psychosocial stress may be one factor that lowers the DHEA and DHEA-S levels. The relationship between prolonged psychological stress and DHEA-S or DHEA levels has been investigated in different ways, but the number of studies is relatively small and some observations of these studies contradict each other. Reduced levels of DHEA and DHEA-S have been reported in association with exposure to prolonged psychosocial stress [16]–[18], but elevated levels has also been reported [19]. Further, some studies do not show any clear association in any direction [20], [21].

The aim of the present study is to investigate whether DHEA and DHEA-S levels differs in individuals that report perceived stress at work compared to individuals who report no perceived stress at work. A unique aspect of this study compared to some of the above mentioned studies is that objective criteria were used to differentiate stressed individuals from non-stressed. Work stressors are a very common reported source of stress, widely studied and shown to cause or contribute to adverse health [1], [3], [22], [23]. If DHEA-S and DHEA levels are reduced in individuals reporting perceived stress at work compared to individuals reporting no perceived stress at work, low DHEA-S and DHEA levels could constitute one link between psychosocial stress, ill health and accelerated ageing.

## Methods

### Ethics Statement

The study was approved by the Regional Ethical Review Board in Gothenburg, Sweden, and was conducted according to the Helsinki Declaration. All participants gave written informed consent before entering the study.

### Participants

The participants in this study were selected from a study of 200 otherwise healthy individuals (50% men) in the age 25 to 50 years aiming to find biological markers of psychological stress. The 200 individuals were recruited from an ongoing longitudinal cohort study at the Institute of Stress Medicine in Gothenburg, Sweden and from advertisements in daily newspapers. Inclusion stratification was initially applied to ensure that participants varied in terms of degrees of perceived stress. Inclusion was therefore based on self-reported level of perceived stress using a single item question from the General Nordic Questionnaire for Psychosocial and Social Factors at Work (QPS Nordic) instrument [24]: “Stress means a situation in which a person feels tense, restless, nervous, or anxious, or is unable to sleep at night because his/her mind is troubled all the time. Do you currently feel this kind of stress?” The response was recorded on a five-point scale varying from “not at all” to “very much.” To ensure that participants varied in terms of degrees of perceived stress, 40 participants (20 men, 20 women) were selected from each of the five stress categories to be included the initial sample of 200 individuals. Before inclusion, the subjects underwent a screening test, including anthropometric measurements and obtaining blood samples to ensure the following exclusion criteria; having a body mass index less than 18.5 kg/m^2^ or over 30 kg/m^2^, high blood pressure, infection, vitamin B-12 deficiency (established by measuring homocysteine), known systemic disease such as diabetes or thyroid disease or known psychiatric disease. Women taking estrogens, nursing, pregnant and postmenopausal women were excluded. Subjects who were taking psychoactive medications or any medications that may affect the hypothalamus-pituitary-adrenal (HPA) axis function were excluded. The inclusion and assessment period was spread across the year for all the different stress-groups. Therefore there were no general differences between the stress groups in terms of when (during which season) they were included and assessed. Among the initial 200 participants, 183 individuals (91 men; 92 women) had data regarding perceived stress at work (see Scoring of perceived stress section). Of these 183 individuals, 172 individuals had serum samples stored in −80°C freezer, available to analyse. Due to the ordinal properties of the scale, the participants were divided into quartiles according to their stress scores and to ensure enough discrepancy in stress levels only the individuals with the lowest and highest perceived stress at work scores (highest and lowest quartiles) were included in the present study. The groups were defined as the non-stressed group (n = 40) and the stressed group (n = 41). Background information on self-reported physical activity intensity the previous three months, educational level (based on occupation) and smoking habits were available.

### Blood Sampling

Blood samples were drawn between 0730 h and 1000 h. The subjects had fasted overnight and were instructed to abstain from hard physical exercise for 24 hours prior to the blood sampling. At arrival, the anthropometry and ECG were performed approximately 30 min prior to the blood sampling. The samples were collected in two different tubes; pre-chilled tubes containing EDTA and serum separator tubes. Plasma and serum were separated by centrifugation, and the samples were stored at −80°C until assayed. For female participants, the blood sampling was conducted between the 5^th^ and 10^th^ day of the menstrual cycle (self-reported mid follicular phase).

### Scoring of Perceived Stress at Work

After blood sampling, the participants answered questionnaires. Perceived stress at work during the past week was measured by using the Stress-Energy (SE) Questionnaire [25]. This questionnaire has been used in several studies on occupational stress [26]–[28] and has been validated for measuring stress at work [29]. In this questionnaire, the participants rate how much they agree to 12 different items using a response scale ranging from *not at all* to *very much* (0–5). Six of these 12 items measure stress level; *stressed*, *tensed*, *under pressure*, *relaxed*, *calm* and *rested* (the response scale were reversed for the three latter items). Thus, they rated to what extent they agreed that the items describe how they felt the previous week at work. The scores from these items were summed up and a mean score was calculated for each participant (Cronbach’s α = 0.956). The mean score of the participants was used in the inclusion procedure in the present study, as described in the selection of participants section, in order to perform a comparison between non-stressed and stressed individuals.

### Hormone Assays

Serum concentration of DHEA was determined using Liquid Chromatography Tandem Mass Spectrometry (LC-MS/MS) method (limit of quantitation, 175 pmol/L), as described in details elsewhere [30]–[32]. Serum concentration of DHEA-S was measured by quantitative electrochemiluminescent immunoassay. Inter-assay coefficients of variation were below 10% for DHEA and below 12% for DHEA-S.

### Statistical Analysis

DHEA levels were not normally distributed (controlled by Kolmogorov-Smirnov test). Logarithmic transformation was therefore performed and log values of DHEA were used in the analyses. Number of men and women in the stressed and non-stressed groups were compared using chi-square test. Scores of perceived stress at work were compared between the non-stressed and stressed group using Mann-Whitney u test. Chi-square tests were performed to check for possible differences between the groups in number of participants who were smokers, had high education level and had a sedentary life style (plausible factors influencing DHEA and DHEA-S). Age and BMI in the two groups were compared using t-test. Pearson’s correlations were computed between DHEA and DHEA-S on one hand and age and BMI on the other hand. Pearson correlation analysis was also performed between DHEA and DHEA-S levels in the stressed and non-stressed group. T-test was used to investigate sex differences in DHEA-S and DHEA levels. To investigate whether there are differences in DHEA-S and/or DHEA levels between stressed individuals and non-stressed individuals, two General Linear Models (ANCOVAs) were performed. DHEA-S and DHEA levels were dependent variables in the two models respectively. Stress group (non-stressed vs. stressed) were entered as predictor. Age was covariate in the models. For all tests, the level of significance was set at p≤0.05, two-tailed. Analyses were conducted with IBM Statistics 20 (SPSS Inc., Chicago, IL, USA).

## Results

Description of the group of individuals that reported stress at work and the group of individuals that reported no stress at work are presented in Table 1.

**Table 1:** Description of the non-stressed and stressed participants.

	Non-stressed (n = 40)	Stressed (n = 41)	*p*-value
Perceived stress at work score	0.77 (0−1.17)	3.50 (3−4.83)	<**0.001**
Number of Men/Women	20/20	15/26	0.320
Age, years	39 (25−50)	40 (25−52)	0.660
BMI, kg/m^2^	23.8 (18−30)	23.4 (19−30)	0.539
DHEA-S, µmol/L	6.3 (2.4−13.3)	4.8 (1.07−8.78)	**0.010** *
DHEA, nmol/L	14.1 (4.37 −36.0)	12.4 (3.92−23.2)	0.348*
High education, %	60%	70%	0.466
Smokers, %	5%	8%	0.675
Sedentary, %	13%	26%	0.250

Mean (range) of the different variables in the non-stress and stressed participants. Number or percentage when indicated. To test for differences between the groups the used test were; Chi-square test, Mann-Whitney test, t-test and General Linear Model statistics (DHEA and DHEA-S).

* p value from analysis in which age was controlled for.

BMI = Body Mass Index, High education = Occupation requiring university education.

### DHEA and DHEA-S Levels in Association to Age and Sex

**Figure 1:**
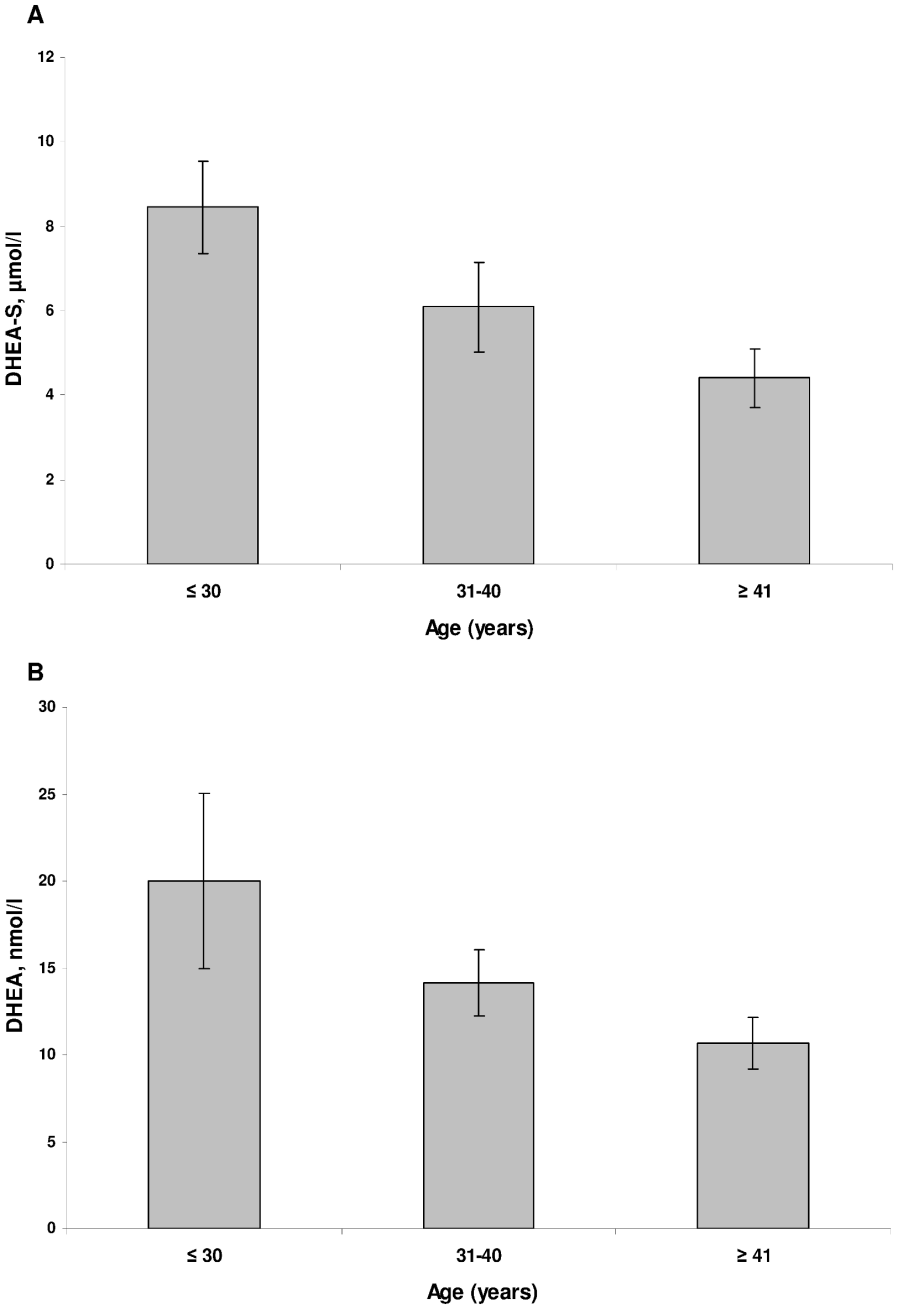
Mean (95% CI) DHEA-S (A) and DHEA (B) levels in different age groups (range 25–52 years).

**Figure 2:**
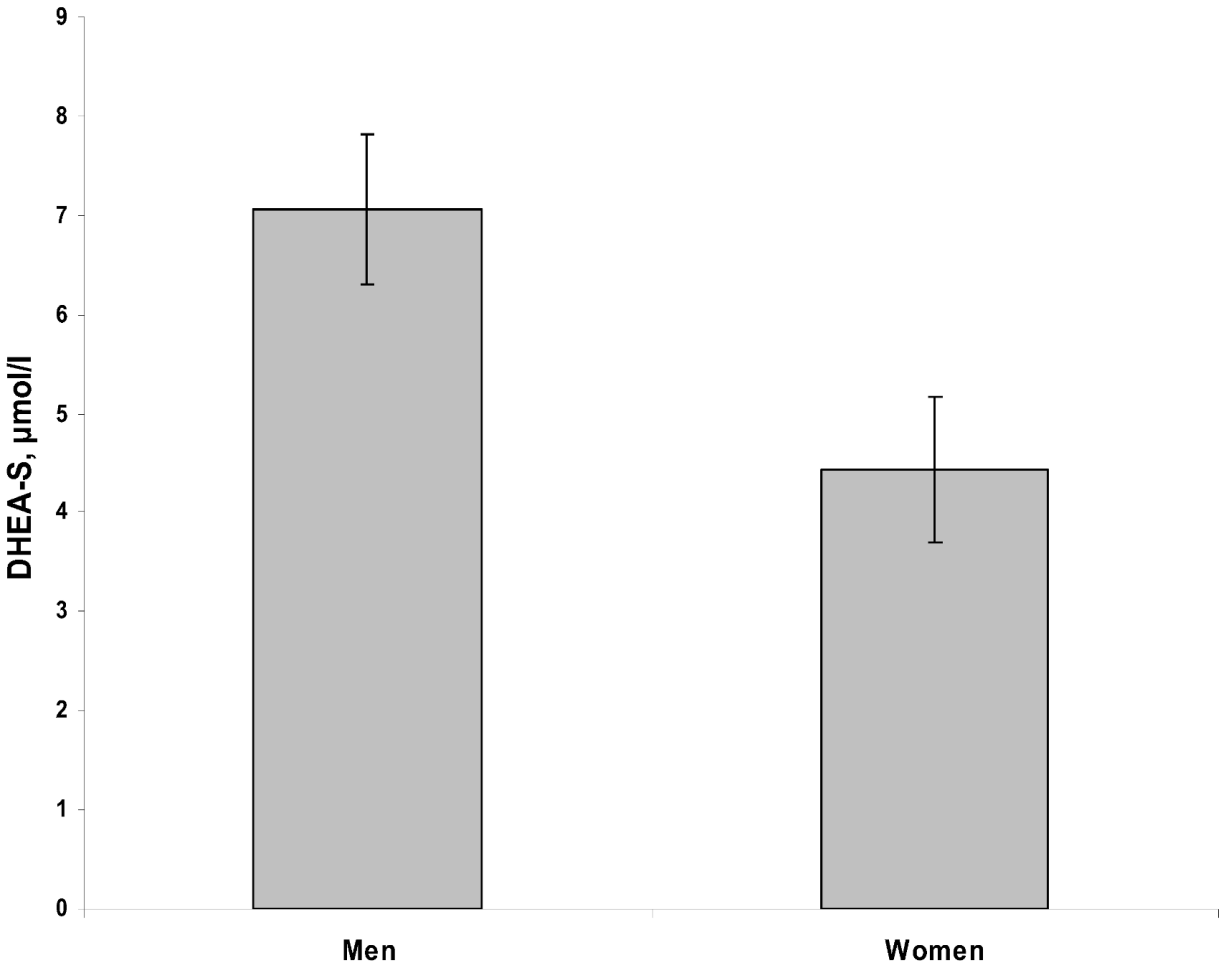
Mean (95% CI) DHEA-S levels in the male and female participants.

DHEA-S and DHEA levels were negatively associated with age (r = −0.50, p<0.001; r = −0.44, p<0.001, respectively). Figure 1 report DHEA and DHEA-S levels in different age groups. DHEA-S and DHEA levels were not associated with BMI (r = 0.01, p = 0.994; r = −0.08, p = 0.457, respectively). DHEA-S levels were significantly higher in men (7.1 µmol/l) than women (4.4 µmol/l) (t = −5.0, p<0.001) (Figure 2) while there was no significant difference in DHEA levels between men (14.1 nmol/l,) and women (12.5 nmol/l) (t = −1.1, p = 0.284). There was a strong correlation between DHEA and DHEA-S levels in both the stressed (r = 0.71, p<0.001) and the non-stressed group (r = 0.65, p<0.001).

### DHEA-S and DHEA Levels in Relation to Perceived Stress at Work

**Figure 3:**
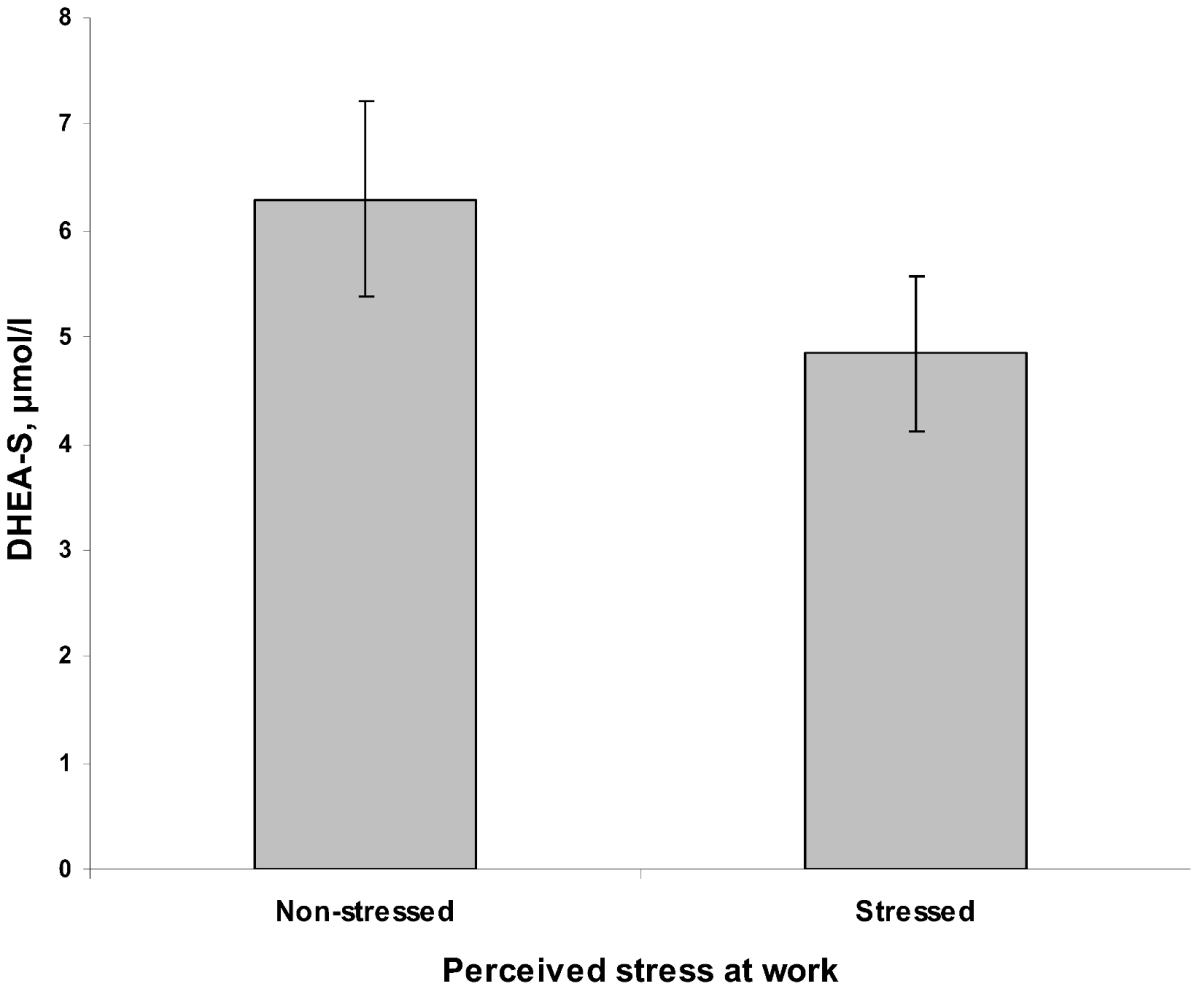
Mean (95% CI) DHEA-S levels in the stressed and the non-stressed individuals.

DHEA-S and DHEA levels in the stressed and non-stressed group are reported in Table 1 and Figure 3. DHEA-S levels were significantly lower [F(1,78) = 6.9, p = 0.010, partial eta squared = 0.08) in the group of individuals reporting stress at work compared to the individuals in the non-stressed group, after controlling for age. Mean DHEA-S level in the stressed group were 23% lower than mean DHEA-S level in the non-stressed group (same pattern in both men and women). There was no difference in DHEA levels between the stressed and the non-stressed group, after controlling for age (p = 0.343).

## Discussion

In this study, DHEA-S and DHEA levels were compared between individuals that reported perceived stress at work and individuals reporting no perceived stress at work. The major finding is that the stressed individuals had markedly lower levels of DHEA-S than the non-stressed individuals. Thus, the stressed individuals had on average 23% lower levels of DHEA-S than the non-stressed individuals. It is known that DHEA-S levels decline with older age [9]; a 20% difference in basal DHEA-S levels corresponds to the difference in the levels seen between 30- and 50-year old individuals. Thus, the results indicate that stressed individuals exhibit DHEA-S levels which are typically observed at age levels above their own. This means that stressed individuals are likely to have lowered anabolic activity, which could in turn cause adverse effects on health and ageing.

In studies on effects of prolonged or chronic stress, like the present study, preferably DHEA-S levels are measured (rather than DHEA levels), since they are more stable and show no or small diurnal variation. Concentrations of DHEA-S are much higher (about 250–500 times) as compared to DHEA, partly related to the fact that DHEA-S has longer half-life and lower clearance than DHEA. DHEA, however, exhibits a diurnal pattern [11]. Diurnal secretion of DHEA exhibits a similar pattern as cortisol secretion [33], thus the levels are highest in the morning after awakening. DHEA level is also more variable because it is more affected by short-time factors such as for example acute psychosocial stress [34]. The present study shows that DHEA levels did not differ between stressed and non-stressed individuals. One plausible reason is that we collected blood samples in the morning and that the morning elevation of DHEA affected the data. Furthermore, since the levels of DHEA are about 300 times lower than the levels of DHEA-S, and since DHEA-S can rapidly convert to DHEA and vice versa [35], this non finding of the relationship between DHEA levels and stress may be of minor biological importance. Future studies measuring DHEA at other times of the day than the morning could reveal different results.

The relationship between prolonged psychological stress and DHEA-S or DHEA levels has been investigated in different ways, but the number of studies is small and there are controversies regarding the results of these studies. Mason et al. (1968) studied the effects on 72 hours avoidance stress in monkeys and found that urinary DHEA levels decreased (20–30%) during the stress exposure period, while cortisol was elevated [36]. Izawa et al (2011) measured perceived stress and salivary levels of cortisol and DHEA before, during and after a two week long teaching practice period in young females [17]. Perceived stress and cortisol levels were increased during the practice period while DHEA was stable. After the practice period, DHEA levels were reduced compared to before and during the practice period. The cortisol awakening curve was also reduced and the authors suggested that the reduction in DHEA levels was caused by the negative feedback control of cortisol (thus reduced ACTH levels). Du et al. (2011) investigated correlations between occurrence of perceived work stressor and levels of plasma DHEA-S in sixty-three bus drivers [20]. They found that DHEA-S levels were higher in drivers who reported concerns related to their salary, but there was no association between DHEA-S levels and the other reported work stressors. Gadinger et al (2011) studied associations between the demand-control-support model and the cortisol to DHEA-S ratio (24 hour urinary cortisol and fasting morning plasma DHEA-S) in 596 employees with management and non-management responsibilities [37]. They found that, in management personnel, higher levels of job demands were associated with lower cortisol to DHEA-S ratios, depending on a positive correlation between job demands and DHEA-S levels. In the non-management personnel there was no association between reported control and demands at work and cortisol to DHEA-S ratio. Kim et al (2010) aimed to test their hypothesis that there are differences in cortisol and DHEA levels (in working subjects) between weekends and workdays, as well as between the beginning of work week and the remaining work week [21]. Cortisol and DHEA levels were measured in saliva samples which were collected 30 minutes after awakening for seven consecutive days in full-time working subjects working Monday to Saturday. The cortisol levels on Sunday were significantly lower than on the workdays. The DHEA levels were lower in the beginning of the work week than on the other days. The authors speculated that these results represented the adrenal response to the upcoming work-related stress. However, it might be difficult to interpret these results because the perceived stress level during the different days was not reported. The type of the stress measures (or lack of stress measure), probably to some extent explains the differences or lack of clarity of the results in the above studies. One strength of our study, compared to the studies described above, is that clearly stressed individuals are compared to individuals that are clearly not stressed. While a mean score of 2.4 on the Stress Energy Questionnaire (possible range is 0–5) has been suggested to constitute a neutral point (showing a neither stressed nor relaxed state) the individuals in the non-stressed group in our study had scores between 0 and 1.17, and the individuals in the stressed group had scores ranging in between 3 and 4.83. We found one study that in which DHEA-S levels were compared between stressed and non-stressed individuals in a similar way to our study [16]. Jeckel et al. (2010) compared salivary DHEA-S levels between female caregivers (seen as chronically stressed) and non-caregivers (seen as non-stressed) in the same age span. The caregivers were significantly more stressed than the non-caregivers and had 32% lower levels of DHEA-S. Thus, our study confirms the results of the study by Jeckel et al. that chronically stressed individuals exhibit lowered DHEA-S levels. Contrary to our study, Lac et al (2012) found higher DHEA-S levels in chronically stressed individuals [19]. They compared salivary DHEA-S in a group of participants that were exposed to bullying at work with levels in a control group not exposed to bullying at work. The participants exposed for bullying at work reported higher stress levels and had higher DHEA-S levels compared to the control group, while there were no differences in any of the cortisol measures. DHEA-S and DHEA have also been found to be elevated in post-traumatic stress disorder (PTSD) [38]–[40]. It is known that DHEA and DHEA-S levels increase during acute psychosocial stress [34], [41], [42], and it could be speculated that in some cases, such as in bullying, a clear acute stress exposure might be present on everyday basis which should result in (repeatedly) acute stressed-induced increase of DHEA and DHEA-S, which might increase the baseline levels. However, during long-term stress, the body has to prioritize expense of the resources and, an optimal “healthy” reaction should be in favour of catabolism and cortisol production, before anabolism and DHEA/DHEA-S production, since in such situations catabolic activity including cortisol is more vital than DHEA/DHEA-S, thus it is more important to address coping with the stressor, than providing protection and regeneration. One explanation is that in such individuals (PTSD patients and persons exposed for bullying) excessive stress has resulted in dysregulation of the HPA axis. Accordingly the regulation of the cortisol and DHEA/DHEA-S production is disturbed. More research is needed to clarify the observed differences between the different studies.

DHEA and DHEA-S have been shown to play an important protective and regenerative role [7], [8]. DHEA has for example been reported to inhibit the production of pro-inflammatory cytokines such as interleukin-6 (IL-6) and tumor necrosis factor (TNF-α) [43], [44], protect low density lipoproteins against peroxidation by free radicals [45], to be protective against the neurotoxic effects of corticosterone [8], [46], and have role in the regeneration of the tissues in the body [47]. Thus, DHEA and DHEA-S have pleiotropic beneficial effects. Reduced levels of DHEA and DHEA-S, as seen in the stressed individuals in the present study, could therefore be associated with adverse effects on health and accelerated ageing, assuming long-term exposure to high levels of perceived stress.

The mechanism behind the lowered DHEA-S levels associated with prolonged psychological stress is unknown. It is known that long-term regulation of DHEA-S levels is modulated by the number of zona reticularis cells and levels of the enzymes 17-hydroxylase, dehydroepiandrosterone sulfotransferase and 3β-hydroxysteroid dehydrogenase within the cells. Ageing is associated with reduced number of zona reticularis cells (which produce DHEA and DHEA-S) [9] and shifted enzymatic activity within the zona reticularis in a way that the capacity to produce DHEA reduce [48]. It is possible that changes in the zona reticularis after long-term stress are comparable to the changes occurring with ageing. Also, during prolonged stress, steroid biosynthesis may be shifted from biosynthesis of adrenal androgens to corticosteroid pathways to ensure maintenance of cortisol production, which is essential during exposure to stressors. Lowered DHEA-S levels in the stressed individuals might also mirror an increased utilization of DHEA and DHEA-S. It should be noted that a small amount of DHEA is produced by the ovary and testis [49], [50].

There are some issues that need to be considered when interpreting the results of this study. It should be noted again that to measuring DHEA in morning, as it was done in this study, is not desirable, as DHEA has a pronounced diurnal rhythm and exhibits morning elevation similar to cortisol, which could affect the observed results. The rationale behind that DHEA were only measured in the morning is that measuring DHEA and DHEA-S was not part of the original plan of the study (with the initial 200 participants described in participants section). Thus, only samples taken in the morning were available for this study. DHEA-S levels, on the other hand, are more stable and show less or no diurnal variation and hence time point of measurement should not affect the results. Thus, DHEA-S should be preferred to measure when effects of long-term stress are studied. Prolonged stress is in this study defined as perceived stress at work during the past week. Work stressors are widely studied and shown to cause or contribute to adverse health [1], [3], [22], [23]. We cannot define our measure of perceived stress at work during one week as a measure of chronic stress, but it can be considered as prolonged stress - compared to an acute stress situation. Although the circumstances during the past week are likely to reflect normal conditions, we cannot know if the observations are reflecting longer exposure to stress, or that the observed inhibited DHEA-S production is an effect of temporary stress exposure at work. In the study by Mason (1968), as little as three days of stress exposure in monkeys reduced DHEA levels by 20–30% [36].

One important question that cannot be answered by the present study is whether, how, and to what extent the DHEA-S and DHEA production could be reversed, normalized and improved. In the study by Mason (1968), DHEA levels returned back to baseline levels during the first six days of recovery. Some studies have used DHEA-S levels as a marker of a positive outcome (increased anabolic activity) of interventions aiming on reducing psychological stress [51]–[55] and some interventions have been shown to be protective. There are studies that strongly indicate that the DHEA-S levels could be improved as an effect of stress-reducing interventions. McCraty studied effects of emotional self-management techniques in 30 healthy individuals [51]. Participants were assessed before and four weeks after receiving training in the techniques. There was significant decrease in perceived stress and in average a 23% reduction in cortisol levels and 100% increase in DHEA-S levels. Also, studies indicated that long-term practice of methods that reduce stress (e.g. meditation and physical activity) may prevent the age-related decline in DHEA-S production [56]–[58]. Thus, the stressed individuals in the present study, which exhibit DHEA-S levels representative of older ages could potentially normalize their DHEA-S levels if the perceived stress at work decrease and suitable resources for recovery are given.

In conclusion, this study indicates that stressed individual have markedly lowered levels of DHEA-S. Stressed individuals exhibit DHEA-S levels that are more typical of levels observed in individuals of older ages. Given the important and beneficial functions of DHEA and DHEA-S, lower levels of DHEA-S, as seen in the stressed individuals in the present study, may be associated with adverse effects on health and accelerated ageing, in circumstances when perceived stress level remain high.
